# Mapping concentrations of posttraumatic stress and depression trajectories following Hurricane Ike

**DOI:** 10.1038/srep32242

**Published:** 2016-08-25

**Authors:** Oliver Gruebner, Sarah R. Lowe, Melissa Tracy, Spruha Joshi, Magdalena Cerdá, Fran H. Norris, S. V. Subramanian, Sandro Galea

**Affiliations:** 1 Harvard T.H. Chan School of Public Health, Department of Social and Behavioral Sciences, Boston, MA, USA; 2 Montclair State University, Department of Psychology, Montclair, NJ, USA; 3 University at Albany, State University of New York, School of Public Health, Department of Epidemiology and Biostatistics, Albany, NY, USA; 4 University of Minnesota Twin Cities, Division of Epidemiology and Community Health, Minneapolis, MN, USA; 5 University of California at Davis, Department of Emergency Medicine, Sacramento, CA, USA; 6 Geisel School of Medicine at Dartmouth, Hanover, NH, USA; 7 Boston University, School of Public Health, Boston, MA, USA

## Abstract

We investigated geographic concentration in elevated risk for a range of postdisaster trajectories of chronic posttraumatic stress symptom (PTSS) and depression symptoms in a longitudinal study (N = 561) of a Hurricane Ike affected population in Galveston and Chambers counties, TX. Using an unadjusted spatial scan statistic, we detected clusters of elevated risk of PTSS trajectories, but not depression trajectories, on Galveston Island. We then tested for predictors of membership in each trajectory of PTSS and depression (e.g., demographic variables, trauma exposure, social support), not taking the geographic nature of the data into account. After adjusting for significant predictors in the spatial scan statistic, we noted that spatial clusters of PTSS persisted and additional clusters of depression trajectories emerged. This is the first study to show that longitudinal trajectories of postdisaster mental health problems may vary depending on the geographic location and the individual- and community-level factors present at these locations. Such knowledge is crucial to identifying vulnerable regions and populations within them, to provide guidance for early responders, and to mitigate mental health consequences through early detection of mental health needs in the population. As human-made disasters increase, our approach may be useful also in other regions in comparable settings worldwide.

Weather related disasters are increasing globally due to a changing climate, and can have far reaching consequences^1^,^2^. In the United States, severe local storms and tropical cyclones were among the most common disasters between 1980 and 2011, together responsible for over $512 billion in damages^3^. Such disasters can have substantial consequences for mental health in the affected population, with posttraumatic stress disorder (PTSD) and depression being among the most commonly reported problems^4^,^5^,^6^. Different patterns of mental health problems have been observed after these events, such that some populations have stably low symptoms with minimal elevations that are limited during the time period during the disaster and its immediate aftermath (resilience), late onset of symptoms (delayed), desisting symptoms (recovery), or stably high symptoms (chronic) of PTSD or depression in the long-term following a disaster^7^.

Several studies to date have explored the individual and socio-ecological factors that shape the likelihood of postdisaster mental health trajectory membership^7^,^8^,^9^,^10^,^11^,^12^. These studies have demonstrated that more severe disaster-related traumatic events (e.g., traumatic injury, bereavement) and stressors (e.g., being displaced, loss of personal belongings and finances), as well as demographic characteristics associated with socioeconomic disadvantage (e.g., female gender, lower income) increase the likelihood of being in a non-resilient trajectory. In contrast, access to social support and community collective efficacy have been associated with resilience^13^,^14^.

It is also possible that *geographic variation* in disaster-related exposures and demographic characteristics influence mental health trajectories. In this vein, researchers have begun to investigate postdisaster mental health using geospatial techniques. For example, studies have shown that greater proximity to the epicenter of a disaster is associated with more severe psychiatric symptoms^15^,^16^, or have mapped disaster-related stress to predict areas in need of services^17^. A more recent study of Hurricane Sandy affected residents in New York City found geographic clusters of higher PTSD and depression in areas closer to the coastline, and that the magnitude and direction of influence of demographic characteristics and disaster-related exposures varied across communities^18^.

To date, the majority of geospatial research on postdisaster mental health has relied on cross-sectional data. Therefore, it is unclear whether the geographic patterning observed reflects mental health responses that unfold in populations over time. Further, prior studies have not examined the factors associated with the geographic concentration of mental health trajectories, leaving open questions about the role that factors such as disaster-related traumatic events and stressors, as well as demographic characteristics play in shaping geographic patterns of risk for mental illness.

In past work focusing on Hurricane Ike in the Galveston, Texas region, we examined geographic variation in resilience from depression and PTSD^19^. However, this study focused only on resilience and did not assess trajectories of delayed symptoms, recovery, or chronic symptoms. In addition, the previous study examined geographic variation in the strength of predictors of resilience, but did not account for whether geographic concentrations of mental health consequences persisted when predictors such as demographic characteristics or disaster exposures were accounted for. It is therefore still unclear whether geographic concentrations in adverse psychiatric health indicators were due to these predictors, or whether they were indicative of unobserved additional risk at the respective locations.

Aiming to build on our previous work, we investigated geographic concentration in elevated risk for the full range of postdisaster trajectories of PTSD and depression symptoms in the same Hurricane Ike affected population, while taking into account potential explanations of the observed trajectories. Hurricane Ike, a Category 2 hurricane, was one of the costliest and most destructive hurricanes in United States history, with over $21 billion of total losses^20^,^21^. Our specific aims were to 1) detect spatial clusters of postdisaster trajectories – resilience, delayed symptoms, recovery, and chronic symptoms – for two mental health indicators, posttraumatic stress symptoms (PTSS) and depression, among Hurricane Ike survivors in Galveston and Chambers counties, TX, to 2) identify predictors for postdisaster mental health trajectories, and 3) to investigate whether spatial clusters of postdisaster trajectories persist after predictors have been controlled for.

## Methods

### Sample

We based our analyses on a longitudinal study with three waves, detailed elsewhere^22^,^23^,^24^. Eligible study participants were aged 18 years or older, and had been living in Galveston County or Chambers County, Texas for at least one month before September 13, 2008, when Hurricane Ike hit. The dataset included 658 respondents at Wave 1 (W1) two-five months after the hurricane, of those respondents, 529 (80%) were still included at Wave 2 (W2) about three months later, and 487 (74%) at Wave 3 (W3) about eight months later^24^. Our complete set of variables without missing data on the locations resulted in a final sample size of 561. Since some respondents reported on similar apartment building addresses (but never from the same apartments within buildings), we introduced a small jitter into the geographic coordinates obtained from these addresses in order to separate them from each other and be able to use this spatial information. We followed the guidelines and recommendations to assure Good Epidemiological Practice (GEP) as defined by the German Society for Epidemiology^25^. The study was therefore conducted in accordance with ethical principles and respected human dignity as well as human rights. After the study was described to the participants at each wave, verbal informed consent was obtained. All study procedures were approved by the institutional review boards of the University of Michigan, Dartmouth College, and Yale University.

### Health indicator variables

For the health indicators in this study, we used trajectory groups for hurricane-related PTSS and depression over all three waves, i.e., resilience, delayed symptoms, recovery, or chronic symptoms, which were calculated in a previous study^26^. Briefly, PTSS were assessed at each wave using the Posttraumatic Stress Disorder (PTSD) Checklist-Specific version (PCL-S)^27^. Questions were asked in reference to the period since the hurricane at W1, and at W2 and W3, the period since the previous interview. The PCL-S consists of 17 items and assessed *DSM-IV-TR* (Diagnostic and Statistical Manual of Mental Disorders, 4th edition, text revision) symptoms of PTSD, such as “repeated, disturbing memories of Hurricane Ike.” Severity scores were calculated as the sum of responses, ranging from 17 to 85, with items rated from 1 (not at all) to 5 (extremely) and a cut-off score of 44 was used, with scores exceeding this threshold being indicative of probable PTSD^27^. Cronbach’s a for the PCL-S scale in the current study ranged from 0.92 to 0.96^26^.

Past-month depressive symptoms were assessed at each wave with the Patient Health Questionnaire (PHQ-9)^28^. Participants were asked if there was a two-week period in the month prior to the interview in which they experienced of nine symptoms (e.g., “feeling down, depressed, or hopeless”) and, if so, how often they were affected by the symptoms. The sum of responses to all items resulted in PHQ-9 scores ranging from 0 to 27, with those who scored 10 or higher being classified into participants who had major depression. Cronbach’s a for the PHQ-9 scale ranged from 0.79 to 0.89^26^.

A four-trajectory solution was then selected for each health indicator (PTSS, depression) based on a group-based mixture model, as detailed in Lowe *et al*.^26^. Each participant was then assigned a probability of being in each trajectory and, based on these values, a most likely trajectory. Trajectories for PTSS or depression were defined as having stably low (resilience), late onset (delayed symptoms), desisting (recovery), or stably high (chronic symptoms) levels over the time of the three waves. For PTSS, 74.9% of participants were classified as being in the resilience trajectory, 5.2% in the delayed symptoms trajectory, 14.7% in the recovery trajectory, and 5.2% in the chronic symptoms trajectory. For depression, 57.9% of participants were classified as being in the resilience trajectory, 10.6% in the delayed symptoms trajectory, 21.6% in the recovery trajectory, and 9.9% in the chronic symptoms trajectory^26^.

### Explanatory variables

Explanatory variables were taken from W1 and are detailed elsewhere^19^,^22^,^23^,^24^,^26^. Demographic variables included age, gender, ethnicity, and highest education completed (Table 1).

We also included variables indicating predisaster trauma exposure, hurricane-related trauma and stressors, emotional reactions during the hurricane, and community-level social assets based on scales that had been shown useful in other studies on Hurricanes Andrew, Katrina, and Ike^29^,^30^.

**For predisaster trauma exposure** we conducted a traumatic events inventory and asked participants (yes/no) whether they had experienced 10 events such as a serious accident or death of family or friends before Hurricane Ike. We then divided the total number of predisaster trauma into three categories: zero to 1, 2 or 3, and 4 or more total traumas before the hurricane. We assessed predisaster probable PTSD with a modified version of the PCL-S in reference to the traumatic event that participants experienced predisaster and designated as the “worst”. The DSM-IV-TR criteria were applied in this context to determine predisaster probable PTSD status. We further applied the PHQ-9 in reference to any 2-week period in their lifetime and classified those who scored 10 or higher on that scale into participants who had predisaster probable major depression. **For hurricane-related trauma**, we asked participants (yes/no) whether they had faced any of the following: physical injury, death of a family member or a close friend, seeing dead bodies, or a family member or close friend injured as a result of Hurricane Ike.

Participants were further asked about six **hurricane-related stressors**, each of which was included as a separate predictor in our study: Whether they (yes/no) were without any resource (food, water, shelter, electricity) for more than one week, had any personal property loss, had any loss of sentimental possessions or pets, had financial loss, had increased demands or relationship problems, or were displaced from home in relation to the hurricane. **Emotional reactions during the hurricane** were assessed with the 4-item STRS (shortness of breath, tremulousness, racing heart, and sweating) checklist^31^. Participants indicated how they felt at the time of the hurricane and following few hours in the aftermath, with a focus on shortness of breath; trembling, shaking, or buckling knees; heart pounding or racing; and sweaty palms or other sweating. We categorized the responses into tertiles with low, medium, and high levels of peri-event emotional reactions.

We assessed **social support** by using the Inventory of Postdisaster Social Support^32^,^33^, which included 11 items, such as “How often did family members or friends express interest and concern in your well-being?” or “…offer or provide you with a place to stay?”, rated from 1 = never to 4 = many times. **Collective efficacy** was applied with a 10-item scale assessing informal social control (for example, “If a group of neighborhood children was skipping school and hanging out on a street corner, how likely is it that your neighbors would do something about it?”), rated from 1 = very unlikely to 5 = very likely and social cohesion and trust (for example, “this is a close knit or unified neighborhood”), rated from 1 = strongly disagree to 5 = strongly agree. Because social support and collective efficacy are conceptualized as functioning at the community level, we first averaged the outcomes for each measure at the census block level and then classified each into either poor (up to median values) or good community social support and collective efficacy (above median values).

### Step-by-step analysis

First, we applied an *unadjusted* spatial scan statistic, which is able to detect spatial clusters of disease^34^,^35^. The spatial scan statistic is implemented in the free software product SaTScan^TM^ and was applied with the multinomial model to identify spatial clusters in the trajectories for PTSS and depression. The spatial scan statistic treats each observation as a case, with each case belonging to one of the trajectory groups for hurricane-related PTSS and depression, i.e., resilience, delayed symptoms, recovery, or chronic symptoms. The scan statistic thereby evaluated whether there were any clusters wherein the distribution of cases was different from the rest of the study region. For example, there may be a higher proportion of cases of recovery or delayed PTSS and a lower proportion of cases with chronic PTSS, while the proportion of cases of PTSS resilience may be about the same as outside the cluster. Relative risk (RR) for each trajectory was thereby calculated as the ratio of the proportions of the number of cases in each category (resilience, delayed symptoms, recovery, or chronic symptoms) out of the total number of cases inside the cluster versus outside. For each location and size of the scanning window, the alternative hypothesis is that there is an elevated risk within the window as compared to outside^35^. Statistical inference for the clusters was evaluated based on a Monte Carlo hypothesis test with 999 replications^34^. We note here that confidence intervals are not available in SaTScan^TM^.

Second, we used multivariable multinomial regression to identify significant predictors of PTSS and depression trajectories using the VGAM package in R^36^.

Third, we applied a predictor-*adjusted* spatial scan statistic (SaTScan^TM^) with the multinomial model of the trajectories for PTSS and depression^34^,^35^. This was to investigate whether geographic concentrations in the trajectories persisted even though we had controlled for the known predictors. Instead of taking the entire sample to scan for spatial clusters in the health indicators, as was done in the *unadjusted* scan statistic, with the predictor-*adjusted* scan statistic, we created a dataset for each of the (categorical) predictors that were found significant for either health indicator in step 2. These datasets represented each categorical predictor (category = 1) together with the given health indicator separately. We then ran SaTScan^TM^ on these multiple datasets simultaneously. This allowed for a separately calculated likelihood function for each dataset (categorical predictor) and helped to detect clusters in one or more predictors and related health indicators while adjusting for different informational content^37^. Inference was again based on 999 Monte Carlo replications.

Fourth, the clusters and point locations were mapped in the geographical information system QGIS^38^,^39^.

## Results

### Spatial clusters of trajectories of posttraumatic stress and depression

**Figure 1:**
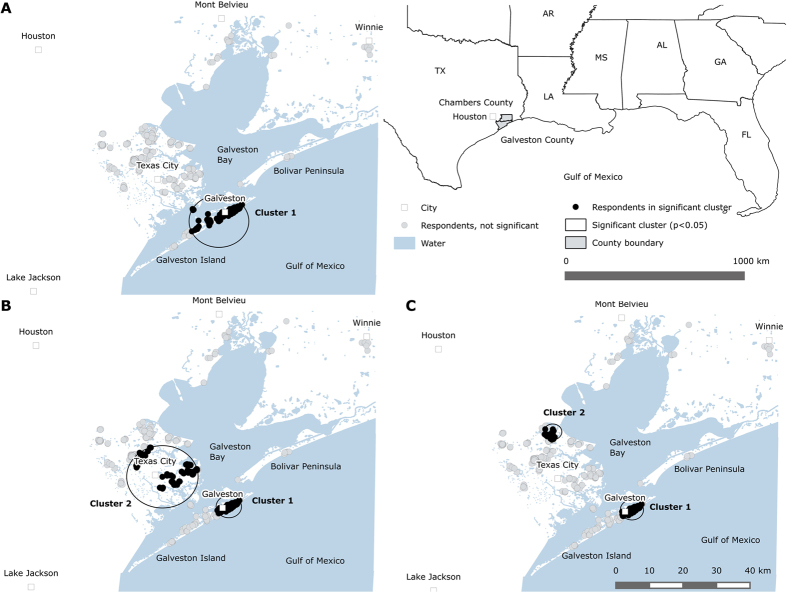
Cluster detection analysis results for PTSS and Depression trajectory groups in Galveston and Chambers counties, TX using the maximum reported spatial window of 40% of the sample population. Map (**A**) shows results from the univariate scan statistic for PTSS, whereas results from multivariable scan statistics are shown for PTSD in (**B**) and for Depression in (**C**). Each dot on the map represents a respondent’s location. Clusters and point locations were mapped in the geographical information system QGIS^38^. Note that the point locations have been altered to preserve confidentiality.

We found one significant (p < 0.01) spatial cluster in *unadjusted* trajectory groups for PTSS (Fig. 1A, Table 2). The cluster had a radius of 8.9 km and included 178 respondents on Galveston Island. The cluster exhibits a region where the risk of chronic PTSS was most dominant in terms of relative risk. The risk of suffering from chronic PTSS was 4.92 times higher inside the cluster compared with outside. The relative risk of recovery from PTSS was 2.51 times greater inside the cluster versus outside; for delayed onset of PTSS, relative risk was 1.54 times higher than outside; and for resilience from PTSS, the risk inside was 0.73 times the risk outside the cluster. Significant spatial clusters of trajectory groups for depression could not be detected.

### Predictors of posttraumatic stress trajectories

Table 3 shows global predictors for PTSS trajectory groups, ignoring respondents’ geographic locations. Significant predictors of delayed PTSS, relative to resilience, were age of 55 years or older, being non-Hispanic Black, and medium or high peri-event emotional reactions. The relative probability of delayed PTSS, versus resilience, was 8.70 times the odds for those in the age group 55 or older compared to 18–34 year olds (95% Confidence Intervals [CI]: 1.60–47.36), 3.34 times the odds for non-Hispanic Blacks compared to whites (95% CI: 1.02–10.88), 8.73 times the odds for those having had medium (versus low) emotional reactions (95% CI: 2.68–28.41), and 14.05 times the odds for those with high (versus low) peri-event emotional reactions (95% CI: 3.93–50.17).

Significant predictors of recovery, relative to resilience, were being non-Hispanic Black, predisaster probable PTSD, loss of sentimental possessions or pets, financial loss as a result of the hurricane, or having medium or high peri-event emotional reactions. The relative probability of recovery, versus resilience, was 3.92 times the odds for non-Hispanic Blacks as compared to whites (95% CI: 1.70–9.01), 5.18 times the odds for those with predisaster probable PTSD compared to those without (95% CI: 2.08–12.92), 4.14 times the odds (95% CI: 2.13–8.04) for those who experienced (versus did not experience) any loss of sentimental possessions or pets, 2.43 times the odds (95% CI: 1.28–4.63) for those who experienced (versus did not experience) a financial loss as a result of Ike, and 3.43 and 11.78 times the odds (95% CIs: 1.57–7.48, 5.47–25.39, respectively) for those with medium or high (versus low) peri-event emotional reactions, respectively.

Significant predictors of chronic PTSS, relative to resilience, were non-Hispanic Black, Hispanic, other ethnicity, predisaster probable PTSD, loss of sentimental possessions or pets, financial loss as a result of the hurricane, and high peri-event emotional reactions. The relative probability of chronic PTSS, versus resilience, was 114.73 times the odds for non-Hispanic Black participants (95% CI: 10.75–1,224.53), 84.61 times the odds for Hispanic participants (CI: 7.98–896.73), and 41.69 times the odds for other non-Hispanic participants (including non-Black and non-white) (95% CI: 1.18–1,477.18) compared to white participants. Those with (versus without) predisaster probable PTSD had 102.88 times the odds (95% CI: 12.57–841.71), those who experienced (versus did not experience) any loss of sentimental possessions or pets had 6.58 times the odds (95% CI: 1.41–30.70), those who experienced (versus did not experience) financial loss as a result of Ike had 15.67 times the odds (95% CI: 2.78–88.35), and those with high (versus low) peri-event emotional reactions had 244.94 times the odds of chronic PTSS, versus resilience (95% CI: 16.76–3,578.79).

### Predictors of depression trajectories

Table 4 shows predictors of depression trajectory groups. Delayed onset of depression, relative to resilience, was significantly associated with age 55 years or older (versus 18–34 years), 4 or more (versus 0–1) traumatic events before Hurricane Ike, predisaster probable major depression, and high (versus low) peri-event emotional reactions. The relative probability for delayed symptoms rather than resilience was 2.54 the odds for those 55 years or older (95% CI: 1.04–6.23), 2.71 the odds for those that experienced 4 or more traumatic events before Hurricane Ike (95% CI: 1.14–6.45), 2.34 times the odds for those that had predisaster probable major depression (95% CI: 1.09–5.02), and 3.2 times the odds for those that had high peri-event emotional reactions (95% CI: 1.44–7.10), relative to their counterparts.

Recovery from depression, versus resilience, was significantly associated with 4 or more (versus 0–1) traumatic events before Hurricane Ike, predisaster probable major depression, financial loss as a result of Ike, and medium and high (versus low) peri-event emotional reactions. The relative probability for recovery, versus resilience, was 2.05 the odds for those that experienced 4 or more traumatic events before Hurricane Ike (95% CI: 1.08–3.92), 2.15 times the odds for those who had predisaster probable major depression (95% CI: 1.20–3.84), 1.69 times the odds for those who had a financial loss due to Ike (95% CI: 1.02–2.81), 1.87 and 2.22 times the odds for those who had medium and high peri-event emotional reactions, respectively (95% CI: 1.06–3.31, 1.19–4.15), relative to their counterparts.

Chronic depression, relative to resilience, was significantly associated with being non-Hispanic Black (versus white), 4 or more (versus 0–1) traumatic events before the hurricane, predisaster probable PTSD and depression, financial loss as a result of Ike, medium and high (versus low) peri-event emotional reactions, and lower collective efficacy in the neighborhood. The relative probability for a chronic depression was 3.55 times the odds for non-Hispanic Blacks (95% CI: 1.44–9.34), 3.27 times the odds for those that had experienced 4 or more traumatic events before Hurricane Ike (95% CI: 1.04–10.26), 2.67 and 3.65 times the odds for those that had predisaster probable PTSD or depression, respectively (95% CI: 1.02–7.01, 1.53–8.71), 3.35 times the odds for those that had a financial loss due to Ike (95% CI: 1.52–7.39), 2.89 and 7.30 times the odds of those that had medium or high peri-event emotional reactions, respectively (C95% CI: 1.05–7.93, 2.92–18.29), and 0.38 times the odds for those that had above median collective efficacy in their neighborhood (95% CI: 0.17–0.83), relative to their counterparts.

### Spatial clusters of predictor-adjusted trajectories of posttraumatic stress

Table 5 shows results of the predictor-*adjusted* spatial scan statistic for PTSS trajectory groups that identified areas of geographic concentration controlling for the significant global risk factors. Cluster 1 was the most likely cluster in terms of significance and was found on Galveston Island. A secondary cluster was found in the hinterland, spanning from Santa Fe to Texas City. The Galveston Island cluster indicated a region wherein chronic PTSS was the most dominant health indicator in terms of relative risk across most of the predictor groups, i.e. subpopulations with a given characteristic. For example, the risk for chronic PTSS was 18.06 times higher for those who were 55 years or older inside the cluster versus those of the same age group outside. The risk for Hispanics was 3.25 times higher, for those that had predisaster probable PTSD 8.00 times higher, for those with any loss of sentimental possessions or pets 7.54 times higher, for those that had a financial loss 4.38 times higher, and for those with medium and high peri-event emotional reactions 11.08 and 3.85 times higher, respectively, inside the cluster versus outside.

For non-Hispanic Blacks, however, delayed onset of PTSS was the most prominent risk and was 10.06 times higher within this cluster as compared to outside the cluster. For other ethnicities (i.e. non-Hispanic, non-black, non-white), the most prominent risk was PTSS resilience being 1.25 times higher inside the cluster compared to outside.

The second cluster in the hinterland indicated a region wherein delayed PTSS was the most dominant health indicator in terms of relative risk across most of the predictor groups, i.e. subpopulations with a given characteristic. Within this cluster, the risk for people 55 years or older was 2.29 times higher for delayed PTSS onset as compared to the same age group outside the cluster. The risk for Hispanics was 8.25 times higher, for those who had predisaster probable PTSD 1.46 higher, for those with a financial loss due to Ike 4.68 higher, and for those with medium or high peri-event emotional reactions 1.60 and 1.76, respectively, higher inside versus outside the cluster. In contrast, the cluster also indicated a region wherein non-Hispanic Blacks had a 1.54 times higher risk for PTSS resilience as compared to outside the cluster.

### Spatial clusters of predictor-adjusted trajectories of depression

Table 6 shows results of the predictor-*adjusted* spatial scan statistic for depression trajectory groups that identified areas of geographic concentration controlling for significant global risk factors. Cluster 1 is the most likely cluster in terms of significance and was found on Galveston Island. A secondary cluster was found around Kemah in the hinterland.

The Galveston Island cluster indicated a region wherein chronic depression was the most dominant health indicator in terms of relative risk across most of the predictor groups, i.e. subpopulations with a given characteristic. For example, the risk for chronic depression was 2.97 times higher for those who were 55 years or older inside the cluster versus those of the same age group outside. The risk for those with 4 or more traumatic events before Hurricane Ike was 3.39 times higher, for those with predisaster probable PTSD 3.20 times higher, for those with predisaster probable depression 2.92 times higher, for those with a financial loss as a result of Ike 2.36 higher, and for those with high peri-event emotional reactions 3.59 higher inside the cluster versus outside.

Furthermore, for non-Hispanic Blacks and those with medium peri-event emotional reactions, the most dominant health indicator was delayed depression onset within this cluster. For non-Hispanic Blacks, the risk was 5.03 times higher within the cluster as compared to outside and those with medium peri-event emotional reactions had a 2.27 times higher risk for delayed onset as compared to outside. In contrast, the cluster also indicated a region wherein those who had above average collective efficacy in their neighborhood had a 1.78 times higher risk of recovery from depression, versus outside the region.

The second cluster around Kemah in the hinterland mostly exhibited a region wherein higher relative risk of resilience from depression was most dominant across all predictor groups, i.e., subpopulations with a given characteristic. The risk of resilience from depression was 1.76 times higher for people 55 years or older inside the cluster versus those of the same age group outside. For non-Hispanic Blacks the risk was 2.05 times higher, for those with 4 or more traumatic events before Hurricane Ike 1.78 times higher, for those with predisaster probable PTSD 2.57 times higher, for those with predisaster probable depression 2.24 times higher, for those with a financial loss as a result of Ike 1.88 times higher, for those with high peri-event emotional reactions 2.94 times higher, and for those with above median collective efficacy in their neighborhood 1.45 times higher inside versus outside the cluster. In contrast, for those with medium peri-event emotional reactions, the risk for chronic depression was most dominant compared to the other trajectories inside the cluster and 1.64 times higher than outside.

## Discussion

Using an unadjusted spatial scan statistic, we detected clusters of elevated risk of chronic posttraumatic stress symptom (PTSS) trajectories, but not depression trajectories, on Galveston Island. We then identified global predictors of membership in each trajectory of PTSS and depression, not taking the geographic nature of the data into account. After adjusting for these predictors in the spatial scan statistic, we noted that spatial clusters of PTSS persisted and additional clusters of depression trajectories emerged.

This is the first study to show spatial clusters of postdisaster mental health symptom trajectories. Spatially varying postdisaster mental health symptoms were found in other studies that suggested a dose-response relationship with more mental health problems in closer proximity to the disaster site^15^,^18^. This may also hold true in our study that found a spatial cluster for PTSS trajectory groups on Galveston Island that was close to the area where Hurricane Ike made landfall. We showed that within this cluster, the relative risk for the delayed PTSS, recovery, or chronic PTSS was higher than outside, while the relative risk for resilience was lower. However, we did not find a significant geographic concentration of risk for any trajectory of depression. This suggests that the predictors for PTSS trajectories were different from those for depression, which might explain why we see a cluster only in the former and not the latter. We investigated predictors of the trajectories in this study and the results were consistent with existing evidence that predictors of PTSS – in this case PTSS trajectories – may differ from those of depression^4^,^5^.

When adjusting our spatial scan statistic for significant predictors in both health indicators, that is, stratifying the population sample by predictor categories and simultaneously scanning for clusters in trajectory groups, we noted that originally detected spatial clusters persisted and additional clusters emerged. There are several possible explanations for the reasons why we found additional clusters although we controlled for the known risk factors. First, clusters of increased risk might be caused by factors specific to the area (e.g. community-level damage). Second, it is likely that clusters represent areas and populations within them in which known individual-level risk factors (e.g., predisaster psychopathology) may interact with other (unknown) individual-level risk factors (e.g. other traumatic life events) together carrying heightened risk for a particular trajectory. Third, a combination of individual- and community-level risk factors (e.g., predisaster psychopathology, higher level of damage) might have contributed to distinct symptom patterns.

For example, in the clusters on Galveston Island (both for PTSS and depression trajectories), the relative risk for chronic health indicators (across most of the predictors) was higher as compared to outside the cluster. A general (non spatial) explanation for this may be that populations at risk (e.g. those with predisaster psychopathology, or those with higher peri-event emotional reactions) are more likely to experience postdisaster mental health problems^4^,^5^,^39^. From our findings, we may assume that in specific situations, i.e., when specific individual- and community-level factors work together, this risk may increase. Galveston City faced flooding and damages to water and wastewater plants, hospitals, and nursing homes, among others^20^. Furthermore, state agency representatives reported that many elderly individuals within evacuation areas did not leave their homes, with some living in unhealthy, mold-damaged dwellings, in tents in their yards or in vehicles^20^. This is consistent with our result that older adults were at increased risk of chronic health indicators in the Galveston Island cluster, i.e. an area heavily affected by the disaster, versus outside of this area.

The secondary cluster for the PTSS trajectories spanning from Santa Fe to Texas City in the hinterland exhibited a geographic region of higher risk for delayed symptoms (across most of the predictors) inside the cluster versus outside. Texas City reported long-term difficulties in the aftermath of the hurricane facing heavy rainfall with water and sewer system outages^40^, which might explain why we see higher risk for delayed onset in this area.

The depression trajectories cluster in Kemah in the hinterland indicated a region of mostly increased likelihood for resilience from depression, which may also be indicative of a complex relationship between individual- and community-level factors. For example, a geographic concentration may emerge in populations with higher than average socioeconomic status residing in an area less affected by the hurricane as compared to other areas. This interpretation could account for our findings, as we noted that residents within the Kemah resilience cluster had above average levels of people with more than a high school degree (92% within the cluster, versus 65% throughout the study area). Furthermore, Kemah and surroundings reported lower damage compared to Galveston Island^40^. We therefore argue that in certain areas, due to a combination of advantageous individual- and community-level factors, the association between known risk factors and psychiatric adversity may be reduced, such that these characteristics are more strongly associated with resilience than in adjacent areas.

In addition, we found that only those with medium peri-event emotional reactions in the Kemah cluster had a higher risk of chronic depression or recovery from depression, which is comparable to other findings that do not take geographic location into account^41^,^42^. Medium peri-event emotional reactions could therefore be a risk factor even among advantaged sub-populations living in less exposed areas.

In summary, our findings suggest that living in areas that were more heavily affected by the disaster were associated with higher risk for chronic or delayed mental health problems. It therefore seems that the influence of geographic location on post-disaster mental symptoms trajectories can be largely explained by disaster exposure of different locations. In addition to different disaster exposures across locations, local socio-ecological conditions seem to determine the risk of mental health symptoms trajectories. These local socio-ecological conditions may be either peoples’ characteristics (e.g. age, ethnicity) varying across space, or socio-ecological factors at the community level, with those communities comprised of better social capital (e.g. collective efficacy) associated with higher risk of resilience or recovery mental health trajectories as compared to other areas with less access to these resources. In this vein, our study elucidated phenomena such as concentrations of individual-level risk or health-promoting factors, or areas wherein several individual-, community- and exposure-related factors combine to determine long-term patterns of mental health. Future work may address the limitations of our study. First, we likely missed some important factors as we still noted clusters in both trajectories after adjusting for known predictors. Additional risk that might stem from situations in which individual- and community level factors work together and mutually enforce each other should therefore be investigated in future studies. Second, we used an unweighted dataset since we were working on the individual level and hence our study is not representative for the entire hurricane-affected population in the area. Future studies may include the background population at risk so that maps would be representative for the entire region. Third, we noted large confidence intervals in the chronic trajectories for PTSS due to low numbers of participants within these categories that should be kept in mind when interpreting our findings.

Notwithstanding these limitations, this is the first study to show that longitudinal trajectories of postdisaster mental health problems may vary depending on the geographic location and the individual- and community-level factors present at these locations. Such knowledge is crucial to identifying vulnerable regions and populations within them before a disaster and may help to provide guidance to improve conditions. In the immediate aftermath of a disaster, early responders may be guided towards those mostly in need, whereas in the longer-term, mental health consequences could be mitigated through early detection of mental health needs in the population. As we currently see an increase in weather related disasters due to a changing climate, our approach may be useful also in other regions in comparable settings worldwide.

## Additional Information

**How to cite this article**: Gruebner, O. *et al*. Mapping concentrations of posttraumatic stress and depression trajectories following Hurricane Ike. *Sci. Rep.* **6**, 32242; doi: 10.1038/srep32242 (2016).

## Figures and Tables

**Table 1:** Descriptive statistics for all variables used in the study.

Variable name	Variable category	*N* (*%*)
PTSS	Resilience	436 (77.7%)
	Delayed onset	24 (4.3%)
	Recovery	78 (13.9%)
	Chronic	23 (4.1%)
Depression	Resilience	342 (61.0%)
	Delayed onset	56 (10.0%)
	Recovery	115 (20.5%)
	Chronic	48 (8.6%)
Age	18–34 years	140 (25.0%)
	35–54 years	199 (35.5%)
	55 years or older	222 (39.6%)
Sex	Women	330 (58.8%)
	Men	231 (41.2%)
Ethnicity	White non-Hispanic	346 (61.7%)
	Black non-Hispanic	83 (14.8%)
	Hispanic	105 (18.7%)
	Other non-Hispanic	27 (4.8%)
Highest level of education completed	Less than high school	69 (12.3%)
	High school degree or equivalent	128 (22.8%)
	More than high school degree	364 (64.9%)
Number of traumatic events before Hurricane Ike	0–1	162 (28.9%)
	2–3	217 (38.7%)
	4 or more	182 (32.4%)
Predisaster probable PTSD	No	491 (87.5%)
	Yes	70 (12.5%)
Predisaster probable major depression	No	443 (79.0%)
	Yes	118 (21.0%)
1 or more hurricane-related trauma	No	500 (89.1%)
	Yes	61 (10.9%)
Without any resource for more than 1 week	No	253 (45.1%)
	Yes	308 (54.9%)
Any personal property loss	No	84 (15.0%)
	Yes	477 (85.0%)
Any loss of sentimental possessions or pets	No	395 (70.4%)
	Yes	166 (29.6%)
Financial loss as a result of Ike	No	376 (67.0%)
	Yes	185 (33.0%)
Increased demands or relationship problems	No	381 (67.9%)
	Yes	180 (32.1%)
Displaced from home as a result from Ike	No	98 (17.5%)
	Yes	463 (82.5%)
Peri-event emotional reactions	Low	332 (59.2%)
	Medium	122 (21.7%)
	High	107 (19.1%)
Social support	Up to median (2.55)	305 (54.4%)
	Above median	256 (45.6%)
Collective efficacy	Up to median (4.1)	301 (53.7%)
	Above median	260 (46.3%)

**Table 2:** Cluster detection analysis results for PTSS and depression trajectory groups in Galveston and Chambers counties, TX, using the maximum reported spatial window of 40% of the sample population and an univariate scan statistic.

Cluster	Radius	LLR	# Obs.	Trajectory group							
				Resilience		Delayed		Recovery		Chronic	
				RR	**%**	RR	%	RR	%	RR	%
PTSS											
1**	8.9km	19.98	178	0.73	61.8	1.54	5.6	2.51	23.6	**4.92**	9.0
2.	9.7km	10.81	114	1.21	90.4	0.17	0.9	0.58	8.8	—	—
Depression											
1	2.9km	9.61	114	0.70	45.6	1.43	13.2	1.20	23.7	2.80	17.5
2	7.9km	7.94	83	1.38	79.5	0.33	3.6	0.55	12.0	0.52	4.8

Notes: LLR = log likelihood ratio, # Obs. = Number of observations in a cluster, RR = relative risk, computed as the ratio of the proportions of the number of cases in each category out of total number of cases inside the cluster versus outside, % = Percentage of cases falling in each group within in a cluster. Significance codes: p < 0.001 ‘***’p < 0.01 ‘**’p < 0.05 ‘*’p < 0.1 ‘.’ Highest RR in bold.

**Table 3:** Multinomial regression model for PTSS trajectory groups in Galveston and Chambers counties, TX.

Reference: PTSD resilience	Delayed onset			Recovery			Chronic		
		95% CI			95% CI			95% CI	
Predictor	OR	LL	UL	OR	LL	UL	OR	LL	UL
18–34 years (ref.)	—	—	—	—	—	—	—	—	—
35–54 years	1.37	0.22	8.33	0.85	0.37	1.96	1.75	0.26	11.70
55 years or older	8.70*	1.60	47.36	2.30	0.97	5.44	5.71	0.77	42.18
Male	0.79	0.27	2.31	0.73	0.38	1.41	0.37	0.06	2.15
White non-Hispanic (ref.)	—	—	—	—	—	—	—	—	—
non-Hispanic Black	3.34*	1.02	10.88	3.92**	1.70	9.01	114.73***	10.75	1,224.53
Hispanic	0.90	0.21	3.92	1.23	0.53	2.85	84.61***	7.98	896.73
Other non-Hispanic	1.60	0.17	15.41	0.84	0.15	4.67	41.69*	1.18	1,477.18
Less than high school degree (ref.)	—	—	—	—	—	—	—	—	—
High school degree or equivalent	0.52	0.14	1.88	0.91	0.34	2.43	1.36	0.15	11.92
More than high school degree	0.30	0.09	1.04	0.77	0.30	1.99	0.41	0.05	3.09
0–1 traumatic events before Hurricane Ike (ref.)	—	—	—	—	—	—	—	—	—
2–3 traumatic events before Hurricane Ike	0.95	0.28	3.23	0.88	0.40	1.89	0.34	0.06	2.14
4+ traumatic events before Hurricane Ike	1.03	0.27	3.96	0.77	0.33	1.79	0.27	0.03	2.56
Predisaster probable PTSD	3.34	0.77	14.43	5.18***	2.08	12.92	102.88***	12.57	841.71
Predisaster probable major depression	1.47	0.42	5.15	1.01	0.47	2.14	1.01	0.17	6.17
1 or more hurricane-related trauma	1.67	0.36	7.81	1.63	0.70	3.79	2.17	0.37	12.62
Without any resource for more than 1 week	1.50	0.54	4.14	1.23	0.65	2.33	1.66	0.33	8.41
Any personal property loss	1.00	0.26	3.85	1.03	0.37	2.88	0.48	0.03	8.83
Any loss of sentimental possessions or pets	2.29	0.77	6.78	4.14***	2.13	8.04	6.58*	1.41	30.70
Financial loss as a result of Ike	1.22	0.39	3.76	2.43**	1.28	4.63	15.67**	2.78	88.35
Increased demands or relationship problems	0.30	0.07	1.28	0.96	0.49	1.88	0.33	0.06	1.72
Displaced from home as a result from Ike	0.49	0.15	1.57	0.47	0.21	1.06	0.36	0.05	2.73
Low peri-event emotional reactions (ref.)	—	—	—	—	—	—	—	—	—
Medium peri-event emotional reactions	8.73***	2.68	28.41	3.43**	1.57	7.48	10.20	0.65	159.88
High peri-event emotional reactions	14.05***	3.93	50.17	11.78***	5.47	25.39	244.94***	16.76	3,578.79
Social support, up to median (ref.)	—	—	—	—	—	—	—	—	—
Social support, above median	1.00	0.35	2.85	1.72	0.90	3.28	3.38	0.75	15.29
Collective efficacy, up to median (ref.)	—	—	—	—	—	—	—	—	—
Collective efficacy, above median	0.56	0.20	1.56	0.60	0.32	1.12	1.21	0.24	6.14

**Table 4:** Multinomial regression model for depression trajectory groups in Galveston and Chambers counties, TX.

Reference: Depression resilience	Delayed onset			Recovery			Chronic		
		95% CI			95% CI			95% CI	
Predictor	OR	LL	UL	OR	LL	UL	OR	LL	UL
18–34 years (ref.)	—	—	—	—	—	—	—	—	—
35–54 years	1.47	0.61	3.57	1.30	0.70	2.43	0.99	0.36	2.77
55 years or older	2.54*	1.04	6.23	1.79	0.94	3.40	2.71	0.97	7.55
Male	0.99	0.52	1.86	0.62	0.38	1.02	0.57	0.25	1.31
White non-Hispanic (ref.)	—	—	—	—	—	—	—	—	—
non-Hispanic Black	2.02	0.89	4.60	1.29	0.64	2.59	3.55*	1.35	9.34
Hispanic	1.22	0.50	2.94	1.26	0.66	2.41	1.23	0.44	3.45
Other non-Hispanic	0.47	0.06	3.90	1.45	0.53	4.02	1.80	0.30	10.64
Less than high school degree (ref.)	—	—	—	—	—	—	—	—	—
High school degree or equivalent	2.14	0.77	5.95	2.22	0.93	5.28	3.11	0.84	11.46
More than high school degree	1.10	0.40	3.02	1.58	0.68	3.65	1.21	0.33	4.37
0–1 traumatic events before Hurricane Ike (ref.)	—	—	—	—	—	—	—	—	—
2–3 traumatic events before Hurricane Ike	1.70	0.75	3.85	1.74	0.96	3.14	2.73	0.95	7.86
4+ traumatic events before Hurricane Ike	2.71*	1.14	6.45	2.05*	1.08	3.92	3.27*	1.04	10.26
Predisaster probable PTSD	0.91	0.32	2.58	1.37	0.66	2.84	2.67*	1.02	7.01
Predisaster probable major depression	2.34*	1.09	5.02	2.15**	1.20	3.84	3.65**	1.53	8.71
1 or more hurricane-related trauma	0.51	0.16	1.65	1.12	0.53	2.35	1.87	0.71	4.98
Without any resource for more than 1 week	1.04	0.55	1.98	1.06	0.66	1.72	0.86	0.39	1.88
Any personal property loss	1.22	0.48	3.09	1.45	0.70	2.99	1.50	0.39	5.85
Any loss of sentimental possessions or pets	1.40	0.71	2.79	1.04	0.61	1.77	1.20	0.54	2.65
Financial loss as a result of Ike	1.90	0.97	3.72	1.69*	1.02	2.81	3.35**	1.52	7.39
Increased demands or relationship problems	0.72	0.35	1.49	1.21	0.72	2.02	1.28	0.57	2.89
Displaced from home as a result from Ike	0.79	0.36	1.74	1.18	0.63	2.22	1.03	0.35	3.04
Low peri-event emotional reactions (ref.)	—	—	—	—	—	—	—	—	—
Medium peri-event emotional reactions	1.93	0.91	4.12	1.87*	1.06	3.31	2.89*	1.05	7.93
High peri-event emotional reactions	3.20**	1.44	7.10	2.22*	1.19	4.15	7.30***	2.92	18.29
Social support, up to median (ref.)	—	—	—	—	—	—	—	—	—
Social support, above median	1.47	0.78	2.79	1.04	0.64	1.69	1.51	0.69	3.34
Collective efficacy, up to median (ref.)	—	—	—	—	—	—	—	—	—
Collective efficacy, above median	0.74	0.39	1.38	0.78	0.49	1.25	0.38*	0.17	0.83

**Table 5:** Cluster detection analysis results for PTSS trajectory groups in Galveston and Chambers counties, TX, using the maximum reported spatial window of 40% of the sample population and the multivariate scan statistic.

	LLR	Predictor group	Trajectory group							
Cluster 1**			Resilience		Delayed		Recovery		Chronic	
Radius			RR	%	RR	%	RR	%	RR	%
3.8 km	72.32	55 years or older	0.66	53.2	1.41	9.7	2.17	25.8	**18.06**	11.3
		non-Hispanic Black	0.37	29.0	**10.06**	19.4	1.10	32.3	5.03	19.4
		Hispanic	0.78	60.0	0.54	2.5	1.42	17.5	**3.25**	20.0
		Other non-Hispanic	**1.25**	100	—	—	—	—	—	—
		Predisaster probable PTSD	0.38	21.4	—	—	0.75	21.4	**8.00**	57.1
		Any loss of sentimental possessions or pets	0.57	41.4	4.80	10.0	1.43	32.9	**7.54**	15.7
		Financial loss as a result of Ike	0.62	44.8	0.36	1.7	1.81	32.8	**4.38**	20.7
		Medium peri-event emotional reactions	0.58	46.2	2.95	15.4	1.99	26.9	**11.08**	11.5
		High peri-event emotional reactions	0.31	16.1	1.47	9.7	1.23	38.7	**3.85**	35.5
Cluster 2*										
10.6 km	37.09	55 years or older	1.15	80.6	**2.29**	12.9	0.24	4.8	0.37	1.6
		non-Hispanic Black	**1.54**	78.6	0.33	3.6	0.56	14.3	0.28	3.6
		Hispanic	1.16	78.6	**8.25**	10.7	0.20	3.6	0.55	7.1
		Other non-Hispanic	0.86	75.0	—	—	—	—	INF	0
		Predisaster probable PTSD	1.30	61.5	**1.46**	7.7	0.52	15.4	0.88	15.4
		Any loss of sentimental possessions or pets	1.76	100	—	—	—	—	—	—
		Financial loss as a result of Ike	1.31	78.0	**4.68**	9.8	0.18	4.9	0.70	7.3
		Medium peri-event emotional reactions	1.05	75.9	**1.60**	10.3	0.80	13.8	-	-
		High peri-event emotional reactions	1.51	58.8	**1.76**	11.8	0.15	5.9	1.51	23.5

Notes: LLR = Log likelihood ratio, RR = relative risk, computed as the ratio of the proportions of the number of cases in each category out of total number of cases inside the cluster versus outside, % = Percentage of cases falling in each group within in a cluster. Significance codes: p < 0.001 ‘***’p < 0.01 ‘**’p < 0.05 ‘*’p < 0.1 ‘.’ Lowest and Highest RR in bold.

**Table 6:** Cluster detection analysis results for depression trajectory groups in Galveston and Chambers counties, TX, using the maximum reported spatial window of 40% of the sample population and the multivariate scan statistic.

	LLR	Predictor group	Trajectory group							
Cluster 1**			Resilience		Delayed		Recovery		Chronic	
Radius			RR	%	RR	%	RR	%	RR	%
3.4km	45.61	55 years or older	0.81	50.0	0.81	10.0	1.10	21.7	**2.97**	18.3
		non-Hispanic Black	0.46	29.0	**5.03**	29.0	0.84	16.1	2.24	25.8
		4+ traumatic events before Hurricane Ike	0.54	30.3	0.68	9.1	1.46	33.3	**3.39**	27.3
		Predisaster probable PTSD	0.32	14.3	0.80	7.1	0.75	21.4	**3.20**	57.1
		Predisaster probable major depression	0.37	17.9	1.17	14.3	1.16	32.1	**2.92**	35.7
		Financial loss as a result of Ike	0.80	40.0	0.50	7.30	1.07	25.5	**2.36**	27.3
		Medium peri-event emotional reactions	0.63	37.5	**2.27**	20.8	1.24	29.2	1.53	12.5
		High peri-event emotional reactions	0.32	14.3	1.41	17.9	0.61	17.9	**3.59**	50.0
		Collective efficacy	0.66	44.4	**1.56**	13.9	1.78	33.3	**1.56**	8.3
Cluster 2*										
2.7 km	35.11	55 years or older	**1.76**	100	—	—	—	—	—	—
		non-Hispanic Black	**2.05**	100	—	—	—	—	—	—
		4+ traumatic events before Hurricane Ike	**1.78**	85.7	—	—	0.27	7.1	0.60	7.1
		Predisaster probable PTSD	**2.57**	85.7	—	—	—	—	0.53	14.30
		Predisaster probable major depression	**2.24**	81.8	—	—	0.61	18.2	—	—
		Financial loss as a result of Ike	**1.88**	85.7	—	—	0.58	14.3	—	—
		Medium peri-event emotional reactions	1.04	57.1	—	—	1.17	28.6	**1.64**	14.3
		High peri-event emotional reactions	**2.94**	100	—	—	—	—	—	—
		Collective efficacy	**1.45**	98.5	—	—	0.49	10.5	—	—

Notes: LLR = Log likelihood ratio, RR = relative risk, computed as the ratio of the proportions of the number of cases in each category out of total number of cases inside the cluster versus outside, % = Percentage of cases falling in each group within in a cluster. Significance codes: p < 0.001 ‘***’p < 0.01 ‘**’p < 0.05 ‘*’p < 0.1 ‘.’ Lowest and Highest RR in bold.
